# Culturally adapted training for community volunteers to improve their knowledge, attitude and practice regarding non-communicable diseases in Vietnam

**DOI:** 10.1186/s12889-024-17938-8

**Published:** 2024-02-03

**Authors:** Zinzi E. Pardoel, Sijmen A. Reijneveld, Robert Lensink, Maarten J. Postma, Nong Thi Thu Trang, Poppy Walton, Khin Hnin Swe, Eti Poncorini Pamungkasari, Jaap A.R. Koot, Jeanet A. Landsman

**Affiliations:** 1 Department of Health Sciences, University of Groningen, University Medical Center Groningen, Hanzeplein 1, Building 3217, Groningen, 9700 RB The Netherlands; 2 Faculty of Economics and Business, University of Groningen, Groningen, The Netherlands; 3 Faculty of Medicine, Department of Pharmacology and Therapy, Universitas Airlangga, Surabaya, Indonesia; 4 Centre of Excellence in Higher Education for Pharmaceutical Care Innovation, Universitas Padjadjaran, Bandung, Indonesia; 5 HelpAge International, Hanoi, Vietnam; 6 HelpAge International, Yangon, Myanmar; 7 Department of Public Health, Faculty of Medicine, Universitas Sebelas Maret, Surakarta, Indonesia

**Keywords:** Community-based approaches, KAP-survey, Community-health volunteers, Community volunteer training, Non-communicable diseases, Culturally adapted training

## Abstract

**Background:**

The burden of non-communicable diseases is becoming unmanageable by primary healthcare facilities in low- and middle-income countries. Community-based approaches are promising for supporting healthcare facilities. In Vietnam, community health volunteers are trained in providing health promotion and screening in a culturally adapted training. This study aims to assess the change in knowledge, attitude and practice regarding NCD prevention and management after a culturally adapted training, and the potential mechanisms leading to this change.

**Methods:**

The Knowledge Attitude and Practice survey was assessed before and after an initial training, and before and after a refresher training (*n* = 37). We used a focus group discussion with community health volunteers (*n* = 8) to map potential mechanisms of the training and applying learned knowledge in practice. Data were collected in the districts Le Chan and An Duong of Hai Phong, Vietnam, in November 2021 and May 2022.

**Results:**

We found that knowledge increased after training (mean = 5.54, 95%-confidence interval = 4.35 to 6.74), whereas attitude and practice did not improve. Next, knowledge decreased over time (m=-12.27;-14.40 to -10.11) and did not fully recover after a refresher training (m=-1.78;-3.22 to -0.35). As potential mechanisms for change, we identified the use of varying learning methods, enough breaks, efficient coordination of time located for theory and practice, handout materials, large group size and difficulty in applying a digital application for screening results.

**Conclusion:**

Culturally adapted trainings can improve knowledge among community health volunteers which is important for the support of primary healthcare in low- and middle-income countries. Using a digital screening application can be a barrier for the improvement of knowledge, attitude and practice and we suggest using an intergenerational or age-friendly approach, with the supervision of primary healthcare professionals. Future research on behavioral change should include additional components such as self-efficacy and interrelationships between individuals.

**Supplementary Information:**

The online version contains supplementary material available at 10.1186/s12889-024-17938-8.

## Background

The global burden of non-communicable diseases (NCDs) is increasing, especially in low- and middle-income countries (LMICs) with rapid economic growth, such as Vietnam, e.g. the share of deaths due to NCDs in LMICs increased from 41% in 1986 to 81% in 2019 [1]. In Vietnam, NCDs have become the leading cause of morbidity and mortality [2]. Major contributors to the NCD burden in Vietnam are cardiovascular diseases, cancer, chronic obstructive pulmonary disease and diabetes. Rates of these diseases are increasing, as a result of strong increases in the risk factors overweight, obesity and hypertension [3]. It is estimated that nearly one in five Vietnamese aged from 30 to 70 years of age, will prematurely die due to one of these NCDs [4]. One of the main issues related to the high burden of NCDs in Vietnam is that most NCD patients do not receive preventive services and diagnoses in time to receive proper treatment [3]. The growing burden of NCDs in LMICs is becoming unmanageable by local and regional primary healthcare facilities [5], and it jeopardizes the ability of healthcare professionals and primary healthcare services to adequately respond to this burden [6].

Community-based approaches are promising practices to support healthcare facilities regarding NCDs, for instance by education and care, provision of social support and collaboration with the healthcare system [7]. At the community level, health outcomes can be optimized within a low resource settings [8, 9]. In Vietnam, community-based approaches are implemented as Intergenerational Self-Help Clubs (ISHCs) [8]. ISHCs are community-based voluntary social organizations, that aim to promote livelihood and wellbeing through a community-based, multifunctional self-help approach. In ISHCs, local community health volunteers (CHVs), i.e., lay people, are chosen among and by members, and in the context of this study trained by HelpAge International Vietnam, an international NGO aiming to improve the roles and capacities of older individuals, in providing health promotion, such as physical health activities, health education, screening for NCDs and raising awareness [8]. The training of the CHVs is aimed to improve knowledge and skills that contribute to awareness, prevention and control of NCDs.

Research has shown that inadequate knowledge of CHVs is a barrier for effective NCD prevention and management at the community level [9]. This is framed in the Theory of Reasoned Action [10], postulating that behavior is partly predicted by attitude and that attitudes are established by behavioral, normative and control beliefs, which are based on knowledge. Improving knowledge of the CHVs by training can improve skills in the prevention and management of NCDs by CHVs. Learned knowledge and skills are lost over time [11, 12] and refresher training can effectively refresh the knowledge and skills of the CHVs [13]. The World Health Organization suggests that CHVs require ‘regular training and supervision’ for community-based approaches to successfully support healthcare [14], which requires culturally suitable training [15]. Moreover, supervision and mentoring of CHVs is essential for improving the quality of service delivery [16, 17]. Many guidelines and recommendations exist for training programs in health promotion for CHVs [18, 19]. However, these trainings are often not adjusted to the culture and context of the target group. As part of the EU-H2020 funded project “*Scaling up non-communicable disease Interventions in South East Asia*” (SUNI-SEA), the training materials of the ISHCs for CHVs have been adjusted to culture and context. such as the incorporation of traditional or cultural habits, using the *Guideline for adaptation of community-based health interventions to culture and context* [20].

Currently, evidence is limited regarding the provision of (ongoing) training of CHVs on the topic of NCDs in LMIC-settings [21], especially culturally adapted trainings. Therefore, this study aims to assess: (1) the change in knowledge, attitude and practice of CHVs regarding NCD-prevention and management immediately after and six months after a culturally adapted training and (2) potential mechanisms leading to the change in knowledge, attitude and practice according to the CHVs.

## Methods

### Study design

We assessed outcomes using two data sources collected in Vietnam, namely: pre- and posttest surveys and a focus group discussion with CHVs who had followed an initial training and refresher training.

### Sample and procedure

The study was conducted in the districts Le Chan and An Duong, of the municipality of Hai Phong in northeastern Vietnam. Hai Phong is located in the Red River Delta region and has a population of over 2 million people. Le Chan is an urban district in which industry is the primary source of livelihood. An Duong is a rural district with agriculture as the primary source of livelihood.

The initial training took four days and focused more on theory and the refresher training took two days and focused mainly on practice. In the trainings the volunteers were informed about the structure of the ISHCs and trained in teaching and communication, health promotion, community empowerment, basic NCD screening and continuum of care. The trainings mostly focused on screening for risk factors for hypertension and diabetes, and on entering the results in a digital application. The topic of NCDs was covered in a more general sense, covering contributing factors like lifestyle, risk factors and family history regarding NCDs. The trainings consisted of lectures, active participation in practicing and games. Per district an initial training (*n* = 95) was organized in November 2021 and a refresher one (*n* = 136) in May 2022. During the initial and refresher training different volunteers participated, some participated only in the initial training and some only in the refresher training. Per training multiple trainers were present, and during the practice parts, the group was divided in smaller groups consisting of six to eight volunteers.

**Fig. 1:**
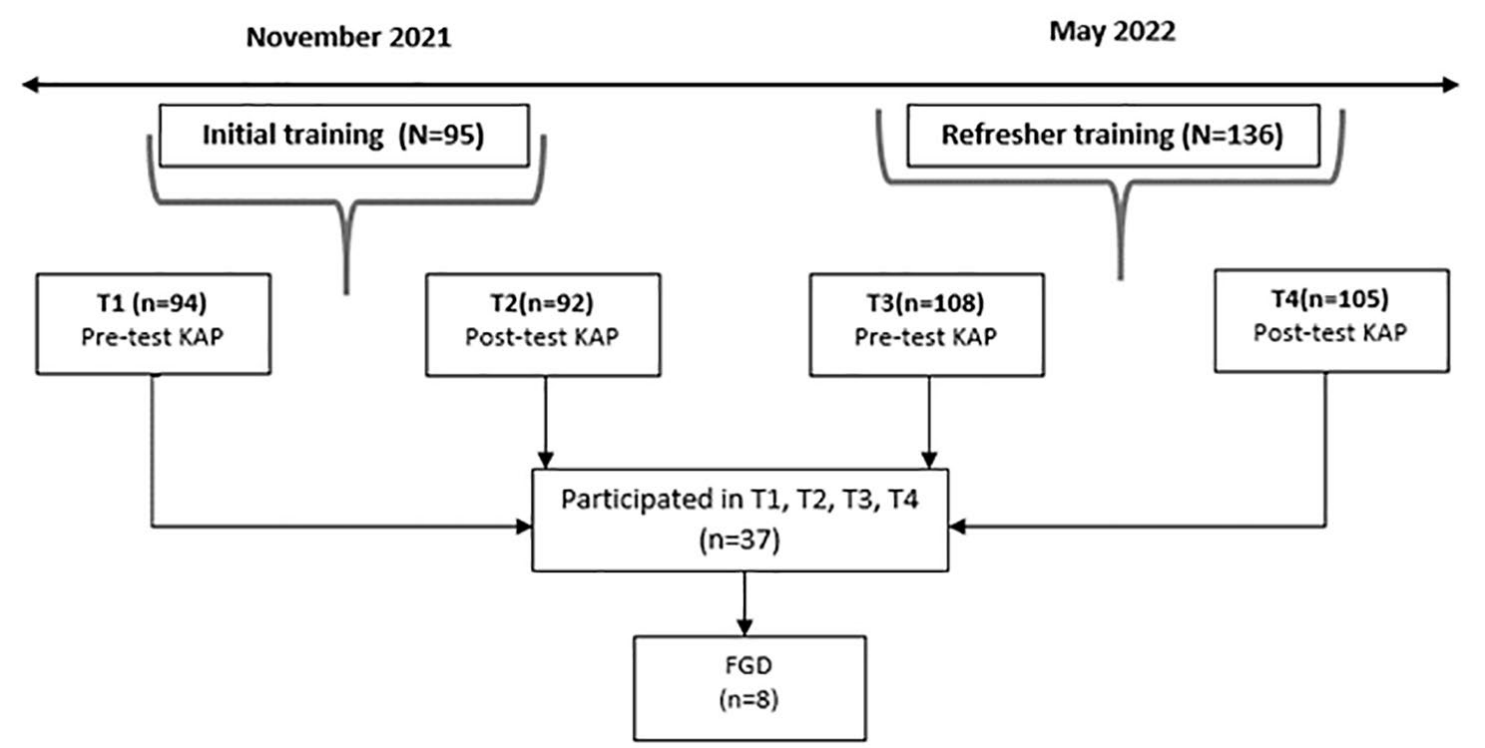
Data collection procedure structured with timeline

Data were collected by HelpAge International Vietnam in November 2021 and May 2022. The first data source consisted of a survey among participants in the training. The survey was based on the validated Knowledge-Attitude-Practice(KAP) framework, i.e., with a structured, validated and standardized questionnaire that measures what is known (knowledge), believed (attitudes), and done (practices) with regard to a specific topic [22–24]. The KAP-survey was developed based on previously validated KAP-surveys covering the same topics and the content of the trainings. In addition, stakeholders involved in the trainings and researchers held four discussions to reach consensus about the final survey. Stakeholders involved in the trainings judged the questions on appropriateness regarding the content of the training and the language level of the volunteers. Researchers involved in the SUNI-SEA project adjusted the questions based on the feedback and relevance for answering the research questions. Data were collected before (*N* = 94) and after (*N* = 92) the initial training in November 2021 and before (*N* = 108) and after (*N* = 105) a refresher training in May 2022, see Fig. 1. CHVs (*n* = 37) were included if they participated four times in the KAP-survey. The study sample of the CHVs and the total volunteer population participating in the KAP-survey at T1, T2, T3, and T4 are approximately similar (see appendix, file 1 for the characteristics of the volunteer population participating in the KAP-survey, per measurement (T1-T4)). The study sample population has a slightly higher mean age and slightly more male participants.

The second data source consisted of a focus group discussion (FGD) among CHVs (*n* = 8) who were selected from the survey sample (*n* = 37) and therefor participated in both trainings and were available and willing to participate in the FGD immediately after the refresher training in May 2022 (see Fig. 1). Moreover, the aim of the FGD was to get in-depth information on the same topics covered in the survey, which helped interpreting and contextualizing the quantitative findings. For manageability, efficiency and quality of the interaction the decision was made to include a maximum of eight participants.

The topic list for the FGD was prepared by the stakeholders involved in the SUNI-SEA project in collaboration with stakeholders involved in the training. Topics were chosen to cover the content of the training. The topic list was circulated two times to evaluate the completeness, comprehensibility and clarity, and discussions were held to validate and develop the final topic list. The FGD was facilitated by a researcher from HelpAge Vietnam and was held directly after the refresher training with seven CHVs and one chairperson of the ISHC, of whom one was male and seven were females from the Le Chan district.

### Measures

For the first aim, we measured the knowledge, attitude and practice in relation to NCD-prevention and management. The KAP-survey consisted of 93 questions and statements, of which 46 questions covered NCDs in general, such as lifestyle, risk factors for NCDs and family history regarding NCDs and 47 questions were focused specifically on hypertension and diabetes (see appendix file 2 for questionnaire). The KAP-survey consisted of 73 knowledge questions (α = 0.78) which covered seven topics, namely: *healthy lifestyle, diabetes and hypertension, NCDs, roles of health volunteer, communication and teaching skills, health promotion, and screening for risk factors*, which were categorized into true or false/don’t know. Eleven attitude statements (α = 0.90), for example: *“Providing health education and promotion has an influence on risk behaviors of NCDs”*, and ten practice statements (α = 0.95), for example: “*I am able to record information properly from screenings for persons at-risk for NCDs*”, were included, which were categorized into positive attitude/practice (answers ≥ mean) and negative attitude/practice (else), based on the mean as the cut-off score. We further collected data on background variables, i.e., *gender*, *district* and *age*.

The second aim (potential mechanisms leading to the change in knowledge, attitude and practice) was assessed with topics in the FGD related to possible influencing factors for the differences in knowledge, attitude, and practice during the training. The performance of the CHVs was discussed (see appendix, file 3 for topic list). Topics covered in the FGD were: 1.Attaining and applying knowledge and skills, such as what helps you to refresh your knowledge? and what helps you to remember the information?, 2. Delivery and content of the training, such as what do you think about the duration of the training?, 3.Content of the training, such as did the content meet your expectations?, 4. Atmosphere and learning environment during the training, such as what is your opinion about the interaction between you and the trainers?, and 5. Practice, such as which contents of the training will be useful in practice?. The FGD was recorded and transcribed in Vietnamese and translated to English. The translation was discussed two times between researchers from HelpAge Vietnam and from the University Medical Center Groningen to reach consensus and ensure cultural and contextual understanding of the translated transcript.

### Analysis and reporting

First, we described the background characteristics of the study sample. Second, we analyzed the increase after training in knowledge, attitude and practice regarding NCD-prevention and management of CHVs by computing pre-post differences and testing these differences using Paired Samples T-tests. A p value of < 0.05 (two-tailed) was considered statistically significant for all associations. Per section (knowledge, attitude, practice) the scores on all items were summed up to compute one variable per section per measurement point (T1, T2, T3, T4). All quantitative measurements and analyses were carried out with IBM SPSS Statistics 28. Third, we assessed the potential mechanisms using content analysis of the FGD data. These data were categorized, grouped, coded and themed. The coding and thematizing was done by one researcher (ZEP) and checked by and discussed with other researchers (NTTT and JAL). Qualitative analyses were carried out with ATLAS.ti 23.

## Results

### Background characteristics

Table 1 shows the results of the descriptive analysis of the background characteristics of the study and sample population of the KAP-survey. In total, 20 (54%) women were included in the study, the average age was 69.5 years, and most respondents lived in Le Chan (70%).

**Table 1:** *Baseline characteristics of the sample population of the KAP-survey*

Background characteristics		n(%)
*Gender*	*Male* *Female*	17(46%) 20(54%)
*Age* ^i^		69.5 *± 6*.2(49–79)
*District*	*Le Chan* *An Duong*	26(70%) 11(30%)
**Total**		37(100%)

^i^ Mean ± stdev (range)

### Change in knowledge, attitude and practice after training

Knowledge was significantly higher after initial training (mean = 5.54; 95%-confidence interval = 4.35 to 6.74) and refresher training (m = 10.49; 95%-CI = 8.47 to 12.50) than before trainings (see Table 2). Knowledge decreased during the six months after the initial training compared to immediately after the initial training (m=-12.27; 95%-CI=-14.40 to -10.14) and decreased after refresher training compared to after initial training (m=-1.78; 95%-CI=-3.22 to 0.35). Last, knowledge decreased from before the refresher training compared to before the initial training (m=-6,73; 95%-CI=-9.59 to -4,78).

**Table 2:** Differences in knowledge, attitude and practice per and between measurement times

KAP	Measurement^i^ (Range of scores)	Mean ± stdev	Between measurements	Mean difference ± SEM^ii^	95% CI
***Knowledge***	*T1 (46–66)*	58.1 ± 4.09	*T1-T2 difference*	5.54 ± 0.59**	4.345 to 6.737
	*T2 (57–70)*	63.6 ± 3.07	*T2-T3 difference*	-12.27 ± 1.10**	-14.400 to -10.140
	*T3 (36–60)*	51.4.8 ± 6.03	*T3-T4 difference*	10.49 ± 0.99**	8.470 to 12.503
	*T4 (55–69)*	61.8 ± 2.77	*T2-T4 difference*	-1.78 ± 0.71*	-3.216 to -0.351
			*T1-T3 difference*	-6.73 ± 0.92**	-9.587 to -4,783
***Attitude***	*T1 (10–50)*	38.1 ± 9.53	*T1-T2 difference*	1.05 ± 1.31	-1.259 to 1.691
	*T2 (11–50)*	39.1 ± 8.89	*T2-T3 difference*	0.00 ± 1.91	-3.888 to 3.888
	*T3 (10–50)*	39.1 ± 10.08	*T3-T4 difference*	-0.11 ± 2.51	-5.199 to 4.983
	*T4 (10–49)*	39 ± 9.75	*T2-T4 difference*	-0.11 ± 2.35	-4.879 to 4.662
			*T1-T3 difference*	1.05 ± 2.20	-3.411 to 5.519
***Practice***	*T1 (12–50)*	41.3 ± 7.98	*T1-T2 difference*	0.78 ± 1.25	-1.750 to 3.317
	*T2 (10–50)*	42.1 ± 7.83	*T2-T3 difference*	-1.43 ± 1.81	-5.103 to 2.238
	*T3 (10–50)*	40.6 ± 8.49	*T3-T4 difference*	-0.51 ± 2.27	-5.116 to 4.089
	*T4 (10–50)*	40.1 ± 11.08	*T2-T4 difference*	-1.94 ± 2.25	-6.510 to 2.618
			*T1-T3 difference*	-0.65 ± 1.91	-4.525 to 3.228

I T1= Before training, T2= After initial training, T3=Six months after initial training, T4= After refresher training

ii SEM= the standard error of the mean. Measures how much discrepancy is likely in the mean compared with the population mean

* Significant at *p*<0.05

** significant at *p*<0.01

No significant differences were found for the practice and attitude scores between the measurement times.

### Possible mechanisms leading to the change in knowledge, attitude and practice according to the CHVs

The CHVs (*n* = 8) mentioned seven potential mechanisms leading to change in knowledge, attitude and practice (see Table 3 for mechanisms and quotes as examples). Potential mechanisms leading to an increase in knowledge, attitude and practice were: (1) Emphasis on practicing, (2) Applying different learning methods, (3) Enough leisure and fun time, (4) Efficient coordination of time located for theory and practice and (5) Handout materials. Potential mechanisms leading to a decrease in knowledge, attitude and practice were: (1) Larger groups, which makes practicing difficult and, (2) Difficulties in learning how to use the application for screening results.

**Table 3:** Findings on potential mechanisms leading to change in knowledge, attitude and practice with quotes

Potential mechanisms	Quote
1. Emphasis on practicing	“*The practical session on screening was the most interesting part, after learning theory, practice helps me understand it clearer*.”
2. Applying different learning methods	“*The use of different learning methods such as listening, speaking, reading and true of false games helps us to remember the information better.”*
3. Enough free and fun time	“*Trainers integrated “tickle methods” during the long training days that helped trainees to stay motivated, such as culture performances and funny games. Also, the breaks gave energy to trainees.*“
4. Efficient coordination of time located for theory and practice	*“For future training I would recommend to spend less time on theory and focus more on practice. One third of the time for theory and two third for practice. Without good coordination 7.5 hours a day is not possible”*
5. Handout materials	*“The handout materials support self-learning of the trainees, to help themselves, their families, and other people with self-prevention at home.”*
6. Group size^*^	*“The training venue was a bit small, especially when playing games. With eight ISHCs it was crowded. Less ISHCs in one course would be more suitable, so that trainers can efficiently train all ISHCs.”*
7. Difficulty in learning how to use a digital screening application	*“The screening application is quite difficult to learn. It would be useful to have an instruction video on the use of the application.”*

*There are +/-100 participants in the training on average

## Discussion

This study showed that a culturally adapted training improved knowledge among community health volunteers (CHVs) after training, whereas attitude and practice did not improve. Six months after training, knowledge decreased, and a refresher training did not fully restore the lost knowledge regarding non-communicable diseases (NCDs) prevention and management. Emphasis on practicing, applying different learning methods, enough leisure and fun time, efficient coordination of time located for theory and practice, and handout materials were mentioned as potential mechanisms for improving knowledge, attitude, and practice. A large group size and difficulties in learning how to apply a digital application for screening results were mentioned as potential mechanisms for a decrease in knowledge, attitude and practice regarding NCD prevention and management.

It is promising that we found an increase in knowledge after training, even if part of this increase was not sustained in the long term. To our knowledge, this is one of the first studies to address the differences between knowledge, attitude and practice regarding non-communicable diseases before, immediately after and six months after a training for CHVs in a low- and middle-income country (LMIC). The findings confirm those of a systematic review about training CHVs in LMICs done by O’Donovan and colleagues (2018), namely that evidence lacked on the provision of ongoing training for NCDs [21]. This study contributes to the literature regarding the outcomes of training for CHVs in low- and middle-income countries.

We found that knowledge improved and attitude and practice did not change. This finding does not correspond with the assumed underlying relationship between knowledge, attitude and practice (KAP) [24] and the Theory of Reasoned Action [25]. The KAP framework theoretically underpins the relationship between knowledge and performance, namely that knowledge influences an individual’s attitude in a positive way and in turn attitude influences practices or changes behavior. An explanation for not finding this relationship could be that behavioral changes are also determined by self-efficacy [26], i.e., beliefs that individuals have based on their expectations of their own abilities and it includes the perceived confidence to conduct a behavior successfully [27]. As CHVs considered it difficult to use the screening application, feelings of low self-efficacy could partly explain the lack of changes in attitude and practice. Moreover, according to the Socio-Ecological approach, behavior is also partly influenced by the context in which it occurs, namely, interrelationships between individuals and the social, physical and policy environment [28]. Research has shown that skills behaviors are strongly influenced by common contextual challenges across LMICs, such as resource availability [29]. The context of the CHVs could have partly influenced the practice outcomes. This finding provides insight into possible core-components of behavioral change after such a training. Further, the CHVs indicated that the group size during the trainings were too large, which made practicing difficult. A study of Thuy and colleagues [30] found improvement of knowledge but no differences in skills after an online course, concluding that online courses are not well suited for teaching skills. An online course does not provide the ability to practice the new learned skills. In accordance, face-to-face training with a large group may also face challenges in providing individualized practice opportunities due to the sheer size of the group, even though the CHVs split up in smaller groups to practice. Not finding a difference in practice after the trainings could partly be explained by lack of time to practice.

We found that knowledge decreased over time and was not fully restored to a similar level after a refresher training. This aligns with general findings that the knowledge attained during a training, quickly decreases [31]. From research, we know that training alone is not sufficient to change CHVs performances and that supervision and mentoring are important for sustaining knowledge and skills [16, 17]. In particular, supervision by primary health care (PHC) professionals seems promising in improving and maintaining knowledge and skills. Supervision and mentoring by PHC professionals can support and improve the performance of CHVs, resulting in improved NCD prevention and management at the community level. This highlights the importance of collaboration between primary health care (PHC) facilities and community-level programs. However, there is limited research on how this supervision and mentoring of PHC professionals should be established. Moreover, the refresher training took two days, compared to the initial training that took four days, and was more focused on practice and less on knowledge attainment. This could partly explain that the knowledge did not restore to the same level after the refresher training compared to after the initial training.

The CHVs indicated that using the digital application for screening results was a barrier for their role as volunteer. Using the application for screening results was considered difficult, which could partly be explained by the age of the CHVs. The average age of the CHVs was approximately 70 years, and from research we know that the uptake and acceptance of digital applications by older adults are rather low [32, 33]. eHealth literacy, i.e., the ability to seek, discover, evaluate and appraise digital health information and apply the acquired knowledge, is rather low among older adults [34], which could explain lower practice scores. A previous study has stated that CHVs need appropriate training to help them acquire (new) digital skills [35]. Moreover, research has shown that an intergenerational approach, i.e., younger volunteers helping older volunteers with the use of the application, or an age friendly application, could contribute to gaining digital skills [36, 37]. Our study contributes to research on eHealth literacy among older adults and on the incorporation of digital applications in trainings for older adults.

### Strengths and limitations

A strength of this study is including the same 37 CHVs in all pre- and post-tests over a period of 6 months. Another strength of the study is that we could add more in-depth to the KAP results, based on the FGD. Finally, this study included a culturally and contextually adapted training, which contributed to knowledge, attitude and practice.

A limitation of this study is the relatively small sample size, which reduced the power of the study. Despite the relatively small sample size, we did find significant associations. And because the study sample population was relatively similar to the total volunteer population at all measurement times, we cautiously expect that findings may have potential for generalization to the broader volunteer population. Another limitation is its use of self-reported data that may have introduced information bias, i.e., giving socially desirable information due to feeling the need to maintain a positive and harmonious relationship with the interviewer or fear of giving wrong answers [38]. However, data were filled in anonymously, reducing the likelihood of such bias. Last, in the KAP-survey, the knowledge part covered 80% of the survey, compared to attitude and practice covering only 20%.Moreover, although the questions were developed based on existing KAP surveys, the content of the training and information collected from various stakeholders who involved in the trainings, they mostly regard self-report data of developed skills, self-efficacy, confidence and knowledge, which may make it difficult to discriminate pre- and post-training practice skills. Additionally, a Hawthorne effect could be present, i.e., that the participation in a training altered CHVs’ behavior simply due to receiving more attention. If so, such an effect will also occur in routine practice, but it deserves further research on which part of the effect is due to the specific contents of the training. This could have contributed to not finding the underlying relationship between knowledge, attitude and practice. Last, the FGD was organized after the refresher training, which may have introduced recall bias. However, the aim of the FGD was to collect ideas and perspectives on mechanisms for change in knowledge, attitude and practice over time. Therefore, it is expected that a recall bias did not affect the data concerning these overall aspects of the training.

### Implications

In this study, we focused on the training of CHVs, which showed to be promising in improving knowledge about NCDs. With an ageing population and growing burden of NCDs, community-based approaches are becoming increasingly important for lowering the burden of healthcare professionals. Future research should concentrate on the outcomes for community members. The outcomes could provide insight for further development and improvement of community-based approaches and trainings for CHVs. Moreover, we suggest developing refresher trainings based on the findings of the KAP. The findings of the KAP can detect the knowledge, attitude and practice gaps on which the training could focus. We expect that by developing the refresher training based on findings of the KAP, levels of knowledge, attitude and practice can increase and be sustained.

We found that a culturally adapted training increases knowledge regarding NCDs. From research, we know that knowledge about health is to a significant degree determined by health perception, and health perception is highly context-dependent [39]. Adapting a community-based training to the context and culture is important to bring out higher knowledge about the prevention and management of NCDs [40]. Culturally adapted trainings are promising as route to reduce the high burden of NCDs but further evidence is needed on reinforced variants. An interesting future design might be to compare the outcomes of a training before and after cultural adaptation. This could provide stronger evidence regarding adapting a training to culture and context.

Our study suggests that researching behavioral changes requires additional components, next to knowledge, attitude and practice, to be included in the research. Based on our findings, we recommend future research on the knowledge and behavioral change of CHVs after training to include components of self-efficacy, interrelationships between individuals and the social, physical and policy environment.

In this study we found that knowledge decreased over time and that a refresher training did not fully restore knowledge. Based on this finding, we suggest creating synergy between PHC and community-based programs. Collaboration between community-based practice and PHC could be beneficial to restore the knowledge of CHVs. When supervised and mentored by PHC professionals, CHVs can be supported and their knowledge and practice can be monitored and updated where needed.

According to the CHVs, learning how to use the application for screening was difficult. Based on this finding and on previous research on age and use of digital applications, we recommend adding more content about the use of digital applications, developing age-friendly applications, using an intergenerational approach, dedicating more time to practice the application during the training, involving PHC professionals in supervision, or considering avoiding digital screening and using paper-based screening for older volunteers.

## Conclusion

We found that a training on non-communicable diseases (NCDs) prevention and management improves the knowledge of community-health volunteers (CHVs), but not their attitudes and practices. The increase in knowledge after this training is promising, even though this increase was not sustained long term. To our knowledge, this is one of the first studies to address the change in knowledge, attitude and practice regarding NCDs before and after a training for community-health volunteers in a low- and middle-income country. Culturally adapted trainings for community-health volunteers can contribute to the provision of NCDs services and are important for synergy between community and primary healthcare to adequately respond to the growing burden of NCDs. Using digital applications among older CHVs can be a barrier, and we suggest using an intergenerational or age-friendly approach with the supervision of primary healthcare professionals. Future research on behavioral change with knowledge, attitude and practice surveys, should include additional components such as self-efficacy and interrelationships between individuals.

### Electronic supplementary material

Below is the link to the electronic supplementary material.

Supplementary Material 1

Supplementary Material 2

Supplementary Material 3

## Data Availability

The dataset(s) supporting the conclusions of this article are available from the corresponding author upon request.

## Abbreviations

e.g.: Exempli gratia, Latin phrase meaning “for example”
CHVs: Community health volunteers
FGD: Focus group discussion
i.e.: Id est, Latin phrase meaning “that is”
ISHCs: Intergenerational Self-Help Clubs
KAP: Knowledge, Attitude, Practice
NCDs: Non communicable diseases
