# Supplementary material for: Culturally adapted training for community volunteers to improve their knowledge, attitude and practice regarding non-communicable diseases in Vietnam
Source: BMC Public Health. 2024 Feb 3;24:364. doi: 10.1186/s12889-024-17938-8 (PMC10837994; doi:10.1186/s12889-024-17938-8)
Supplement: Supplementary file 1 — Supplementary Material 1 [file 12889_2024_17938_MOESM1_ESM.docx]

**Supplementary file 1** Table A1 Characteristics of volunteer population of the KAP-survey

| **Background characteristics** | | **T1 n(%)** | **T2 n(%)** | **T3 n(%)** | **T4 n(%)** |
| --- | --- | --- | --- | --- | --- |
| *Gender* | *Male  Female* | 38(40%)  56(60%) | 32(35%)  60(65%) | 41(38%)  67(62%) | 38(36%)  67(64%) |
| *Age^i^* | | 66.9*±6.9*  (41-81) | 66.2*±7.5*  (41-84) | 66.3*±9.5*  (24-87) | 65.8*±9.9*  (49-79) |
| *District* | *An Duong*  *Le Chan* | 32(34%)  62(66%) | 28(30%)  64(70%) | 44(41%)  64(59%) | 41(39%)  64(61%) |
| **Total** |  | 94(100%) | 92(100%) | 108(100%) | 105(100%) |
