# Supplementary material for: Culturally adapted training for community volunteers to improve their knowledge, attitude and practice regarding non-communicable diseases in Vietnam
Source: BMC Public Health. 2024 Feb 3;24:364. doi: 10.1186/s12889-024-17938-8 (PMC10837994; doi:10.1186/s12889-024-17938-8)
Supplement: Supplementary file 2 — Supplementary Material 2 [file 12889_2024_17938_MOESM2_ESM.docx]

**Supplementary file 2 Knowledge, Attitude and Practice survey**

**A. Knowledge on Healthy Lifestyle, NCDs risk factors, prevention, complications, and symptoms**

|  | **Question** | **TRUE** | **FALSE** | **Don't know** |  |  |
| --- | --- | --- | --- | --- | --- | --- |
|  | **Healthy Lifestyle** |  |  |  |  |  |
| 1 | Self-care with regular health checks is important for a healthy lifestyle | x |  |  |  |  |
| 2 | Steamed/boiled vegetables are healthier than fried vegetables | x |  |  |  |  |
| 3 | People should at least have 150 minutes of moderate physical activity per week to prevent NCDs | x |  |  |  |  |
| 4 | The maximum salt intake per day is < 5g (one teaspoon) | x |  |  |  |  |
| 5 | The recommended sugar intake per day is <15g (three teaspoons) | x |  |  |  |  |
|  | **Diabetes & hypertension** |  |  |  |  |  |
| 6 | Diabetes is a heredity disease, a person can only get it if it runs in the family |  | x |  |  |  |
| 7 | Hypertension can be transmitted from one person to another |  | x |  |  |  |
| 8 | Diabetes patients with severe infection and /or foot ulcer must be referred to health facilities | x |  |  |  |  |
|  | What are common complications of diabetes? (true/false per option) |  |  |  |  |  |
| 9 | a. Heart failure | x |  |  |  |  |
| 10 | b. Foot damage | x |  |  |  |  |
| 11 | c. Hair loss |  | x |  |  |  |
| 12 | d. Depression |  | x |  |  |  |
| 13 | e. Skin changes colour |  | x |  |  |  |
|  | What are common complications of hypertension? (true/false per option) |  |  |  |  |  |
| 14 | a. Heart failure | x |  |  |  |  |
| 15 | b. Kidney failure | x |  |  |  |  |
| 16 | d. Hair loss |  | x |  |  |  |
| 17 | e. Blurred vision | x |  |  |  |  |
| 18 | f. Skin changes colour |  | x |  |  |  |
|  | What are symptoms of diabetes? (true/false per option) |  |  |  |  |  |
| 19 | a. Increased thirst | x |  |  |  |  |
| 20 | b. Watery eyes |  | x |  |  |  |
| 21 | c. Frequent urination | x |  |  |  |  |
| 22 | d. Extreme hunger | x |  |  |  |  |
| 23 | e. Blurred vision | x |  |  |  |  |
| 24 | f. Pain in stomach |  | x |  |  |  |
|  | What are symptoms of hypertension? (true/false per option) |  |  |  |  |  |
| 25 | a. Severe headache | x |  |  |  |  |
| 26 | b. Fatigue |  | x |  |  |  |
| 27 | c. Chest pain | x |  |  |  |  |
| 28 | d. Pain in stomach |  | x |  |  |  |
| 29 | e. Vision problems | x |  |  |  |  |
|  | **General knowledge on NCDs** |  |  |  |  |  |
| 30 | In Vietnam, the current burden of NCDs is greater than infectious diseases plus maternal, neonatal, and nutrition conditions | x |  |  |  |  |
| 31 | A FINDRISC score above 15 indicates high risk for diabetes, and implicates that the person should be referred to the health facility |  |  |  |  |  |
| 32 | At the time of blood pressure check, people should be in a lying position |  | x |  |  |  |
| 33 | Participants in screening need to be asked about the history of non-communicable diseases that they have had/are having | x |  |  |  |  |
| 34 | Only people who are over 60 years old are at risk of getting NCDs |  | x |  |  |  |
| 35 | Four main behavioural risk factors of NCDs are tobacco use, physical inactivity, harmful use of alcohol, and unhealthy diets | x |  |  |  |  |
| 36 | A fasting blood sugar level from 3.9 - 5.6 mmol/L is normal | x |  |  |  |  |
| 37 | Patients with a blood pressure more than 200/100 mm Hg must be referred urgently | x |  |  |  |  |
| 38 | It is recommended that men drink no more than 2 units and women no more than 1 unit of alcohol a day | x |  |  |  |  |
| 39 | Diabetes can be transmitted from one person to another |  | x |  |  |  |
| 40 | Hypertension is a heredity disease, a person can only get it if it runs in the family |  | x |  |  |  |
| 41 | When a person has diabetes or hypertension, taking medicine is sufficient |  | x |  |  |  |
| 42 | Hypertension can be cured |  | x |  |  |  |
| 43 | Taking medicine when prescribed is important for healthy living | x |  |  |  |  |
| 44 | Eating five or more servings of fruits and vegetables a day can contribute to reducing risk for NCDs | x |  |  |  |  |
| 45 | Diabetes can be cured |  | x |  |  |  |

**B. Knowledge on training contents**

|  | **Question** | **TRUE** | **FALSE** | **Don't know** |  |
| --- | --- | --- | --- | --- | --- |
|  | **Roles of health volunteers** |  |  |  |  |
| 46 | I work with ISHC Management Committee to conduct the health communication session in ISHC monthly meetings and other occasional events to ISHC members and community members according to ISHC annual plan | x |  |  |  |
| 47 | I conduct basic health screening to all ISHC members, homecare clients, and needy people in the communities | x |  |  |  |
| 48 | I conduct regular health check-ups for ISHC members and community people |  | x |  |  |
| 49 | I provide proper medication to ISHC members and community people in need |  | x |  |  |
| 50 | I support ISHC members and community people to access proper health services by coordination with primary health care unit in the community (village, commune health station, district health center) | x |  |  |  |
|  | **Communication and teaching skill** |  |  |  |  |
| 51 | Before organizing health information sessions, the facilitators and speakers should introduce themselves as well as the topic and the length of time for the communication session | x |  |  |  |
| 52 | Facilitators should give time for participants to ask questions | x |  |  |  |
| 53 | People can learn the most by seeing and hearing |  | x |  |  |
| 54 | There should be funny games for the participants during health information session | x |  |  |  |
| 55 | If the health communication session is less than 30 minutes, there is no need to have a review session for participants |  | x |  |  |
|  | **Health Promotion** |  |  |  |  |
| 56 | A person with a disability or chronic disease can continue to live a healthy life | x |  |  |  |
|  | A healthy lifestyle can be achieved through (true/false per option) |  |  |  |  |
| 57 | a. Stop smoking | x |  |  |  |
| 58 | b. Stop use alcohol | x |  |  |  |
| 59 | c. More physical exercise | x |  |  |  |
| 60 | d. A healthy diet | x |  |  |  |
| 61 | e. Good self-care | x |  |  |  |
|  | Types of exercises that you should do within a week (true/false per option) |  |  |  |  |
| 62 | a. Aerobic exercises (total 2.5 hours for light exercise or 1.5 hours for intense exercise) | x |  |  |  |
| 63 | b. Muscle strengthening exercises (2-3 times a week) | x |  |  |  |
| 64 | d. Balance and flexibility (2-3 times a week) | x |  |  |  |
| 65 | People with limited mobility should do exercise 45-60 minutes every day | x |  |  |  |
|  | **Screening risk factors** |  |  |  |  |
| 66 | Screening risk factors can prevent and control NCDs | x |  |  |  |
| 67 | Selection of persons to be screened is > 40 years | x |  |  |  |
| 68 | Screening processes include the use of questionnaire and taking measurement | x |  |  |  |
|  | Necessary equipment for basic screening are (true/false per option) |  |  |  |  |
| 69 | a. Weight scale | x |  |  |  |
| 70 | b. Measuring tape | x |  |  |  |
| 71 | c. Blood pressure monitor | x |  |  |  |
| 72 | d. BMI chart | x |  |  |  |
| 73 | e. Tablet | x |  |  |  |

**C**. **Attitude**
The following questions can be answered on a scale of 1(totally disagree) to 5 (totally agree)

|  | **Question** | **Strongly disagree** | **Disagree** | **Neutral** | **Agree** | **Strongly agree** |
| --- | --- | --- | --- | --- | --- | --- |
| 1 | A good diet, and physical exercise contribute to healthy lifestyles |  |  |  |  |  |
| 2 | Alternative treatments (traditional medicines, herbs, etc.) are better than prescribed medicines |  |  |  |  |  |
| 3 | Providing health education and promotion has an influence on risk behaviours of NCDs |  |  |  |  |  |
| 4 | The ISHC has a positive influence on healthy lifestyles |  |  |  |  |  |
| 5 | Collaboration between ISHCs and health facilities is important to prevent and manage NCDs |  |  |  |  |  |
| 6 | It is important to measure height, weight, body mass index, abdominal circumference and blood pressure in ISHC |  |  |  |  |  |
| 8 | I feel comfortable to preform activities such as physical exercise and health promotion in the community group |  |  |  |  |  |
| 9 | I feel comfortable to do screening measurements |  |  |  |  |  |
| 10 | I can motivate people to live a healthy lifestyle |  |  |  |  |  |
| 11 | I am satisfied with my role as a health volunteer |  |  |  |  |  |

**D. Practice**The following questions can be answered on a scale of 1(totally disagree) to 5 (totally agree)

|  | **Question** | **Strongly disagree** | **Disagree** | **Neutral** | **Agree** | **Strongly agree** |
| --- | --- | --- | --- | --- | --- | --- |
| 1 | I am able to record information properly from screening for persons at-risk for NCDs |  |  |  |  |  |
| 2 | I am able to follow-up on persons at risk for NCDs properly |  |  |  |  |  |
| 3 | If the screening results are indicating symptoms of NCDs, I refer them to the local health facility |  |  |  |  |  |
| 4 | I feel comfortable to mobilise people to join activities (i.e., physical exercise, health promotion talks) at the community-based program |  |  |  |  |  |
| 5 | I know how to screen for diabetes |  |  |  |  |  |
| 6 | I know how to screen for hypertension |  |  |  |  |  |
| 7 | I know when I should follow-up on a patient |  |  |  |  |  |
| 8 | I know when a person is at risk or has diabetes based on screening results |  |  |  |  |  |
| 9 | I know when a person is at risk or has hypertension based on screening results |  |  |  |  |  |
| 10 | I have educated person who has unhealthy life style |  |  |  |  |  |
