# Supplementary material for: Culturally adapted training for community volunteers to improve their knowledge, attitude and practice regarding non-communicable diseases in Vietnam
Source: BMC Public Health. 2024 Feb 3;24:364. doi: 10.1186/s12889-024-17938-8 (PMC10837994; doi:10.1186/s12889-024-17938-8)
Supplement: Supplementary file 3 — Supplementary Material 3 [file 12889_2024_17938_MOESM3_ESM.docx]

**Supplementary file 2 Focus group discussion: Topic list**

Focus: which barriers and facilitators do cadres experience to implement obtained knowledge and skills in practice?

**Opening of the meeting**

Moderator and assistant of the focus group discussion will introduce themselves, explain the goals of the discussion, thank the participants for taking the time and explain the ground rules. Goals of the discussion are to answer the following questions:

- How did the training improve/change your knowledge, attitude and practice?
- What is needed to keep the knowledge, attitude and practice on the same level?

The ground rules are:

- Discussion will be recorded
- Full confidentiality
- There are no right or wrong answers
- You don't need to agree with others, but you must listen respectfully as others

Small introduction of group members to establish a sense of community and start with a opening question, such as: How was your day? Did you arrive well? How was your way here?

**Introduction**

- How did you apply your new knowledge and skills?

1. **Key questions**

What do you think about the consistency of your attained knowledge, attitude and practice?

- What helps you remember the information?
  - Are there cues/mnemonics or pictures in your head that you use
    - Did you attain those cues/during the training? If yes how, why, when?
    - Or did you make them up yourselves
- What do you think would have helped you to remember the new information better?
- What would help you to refresh your knowledge (practical + theoretical)
- What would help you in your work environment
- What do you think about the possibility of a recap training?

**Delivery and planning of the training**

What do you think about the delivery of the training?

- What do you think about the duration of the training?
- What do you think about the speed of information during the training?
- What do you think about the size of the group?
- What do you think about the amount and length of the breaks during the training?
- Do you have any suggestions to improve the delivery and planning of the training?

**Content**

What do you think about the content of the training?

Did the content of the training meet your expectations? What would you change?

- What do you think about the amount of theoretical training?
- What do you think about the amount of practical training?
- What do you think about the level of difficulty of the content?
- What did you find interesting?
- What did you not find interesting? What bored you?
- What information was new to you?
- Was the material personally meaningful to you?
- Did you miss information that you would have liked to learn about?
- Did you do further research into the topic after the training? If yes: how? If not: why/
- What are your suggestions to improve the content delivered in this training?

**Collaboration and motivation**

How was the learning environment and group atmosphere during the training?

- How did you feel during the training?
- What was your level of arousal during the training?
- What is your opinion on the interaction between you and the trainers?
- What is your opinion on the interaction between you and the other volunteers?
  - (e.g., ask for amount, quality of interaction)
- How did you feel when asking questions?
  - Did you ask all the questions you had?
  - How satisfied were you with the answers during the Q&A session?
- What motivated you to go to the training?
  - (e.g., was it intrinsic or extrinsic motivation
- What demotivated you to go to the training?
  - (e.g., the distance to travel to the training?)
- What motivated you to pay attention and learn during the training?
  - What was a pitfall for your motivation?
- What kept you from paying attention during the training?

What feedback (compliments and tips) would you like to give to the facilitators?

**Community meetings / Working practice**

How well did the training prepare for working at the community meetings?

- Which uncertainties do you think you will face while working at the community meetings?
- How appropriate was the amount of practical training?
- How appropriate was the amount of theoretical training?
- Which contents of the training will be useful in practice? Which were not?
- What do you think will facilitate you as a health volunteer?
- What feedback (compliments and tips) would you like to give to the facilitators?
  - What else would you want to learn to have better work in practice?
  - What would you want to modify about the training course?

1. **Summary**

Give summary of everything discussed

1. **Questions at the end**

Of all the things we discussed, what is the most important to you?
